# A Leishmania secretion system for the expression of major ampullate spidroin mimics

**DOI:** 10.1371/journal.pone.0178201

**Published:** 2017-05-23

**Authors:** Todd A. Lyda, Elizabeth L. Wagner, Andre X. Bourg, Congyue Peng, Golnaz Najaf Tomaraei, Delphine Dean, Marian S. Kennedy, William R. Marcotte

**Affiliations:** 1 Department of Genetics and Biochemistry, Clemson University, Clemson, South Carolina, United States of America; 2 Department of Materials Science and Engineering, Clemson University, Clemson, South Carolina, United States of America; 3 Department of Bioengineering, Clemson University, Clemson, South Carolina, United States of America; Chang Gung University, TAIWAN

## Abstract

Spider major ampullate silk fibers have been shown to display a unique combination of relatively high fracture strength and toughness compared to other fibers and show potential for tissue engineering scaffolds. While it is not possible to mass produce native spider silks, the potential ability to produce fibers from recombinant spider silk fibers could allow for an increased innovation rate within tissue engineering and regenerative medicine. In this pilot study, we improved upon a prior fabrication route by both changing the expression host and additives to the fiber pulling precursor solution to improve the performance of fibers. The new expression host for producing spidroin protein mimics, protozoan parasite *Leishmania tarentolae*, has numerous advantages including a relatively low cost of culture, rapid growth rate and a tractable secretion pathway. Tensile testing of hand pulled fibers produced from these spidroin-like proteins demonstrated that additives could significantly modify the fiber’s mechanical and/or antimicrobial properties. Cross-linking the proteins with glutaraldehyde before fiber pulling resulted in a relative increase in tensile strength and decrease in ductility. The addition of ampicillin into the spinning solution resulted in the fibers being able to inhibit bacterial growth.

## Introduction

Silk is a natural composite fiber containing protein at its core [1] and has been used in a wide array of applications from traditional textiles to medical devices. While most of the silk used within commercial applications has been traditionally collected from silkworms, other naturally produced silk fibers, such as those produced by spiders, have been shown to have relatively superior and unique mechanical properties.

Spiders can actually produce up to seven different silk fiber types that are each specialized for specific applications within their natural environment [2]. This variety in mechanophysical properties makes spider silk fibers attractive for new biomaterials development [3]. Dragline silk, which makes up the reels within orb weaver webs, is the most characterized spider fiber and has been shown to display specific tensile characteristics that are higher than the properties of steels and other man-made materials [4,5].

The core of a dragline fiber is composed almost exclusively of the major ampullate spidroins 1 and 2 (MaSp1 and MaSp2) that self-assemble into a fiber [6,7]. Spidroins remain concentrated in solution within the duct before being drawn through the spinneret as a solid fiber. It has been postulated that the N-terminal and C-terminal domain regions (NTD and CTD, respectively) of MaSP1 and MaSP2 aid in maintaining the solubility and in regulating the self-assembly process while the central block repeat units, that are flanked by the NTD and CTD, contribute largely to the overall strength of the molecule[2,8,9].

A limiting factor to realizing commercial products made from native spider silk has been the intractability of harvesting silk directly from spiders [10]. One method to incorporate spider silks into commercial products is to fabricate fibers from recombinantly-produced spidroins or spidroin-like proteins. This approach, however, is dependent upon successful cloning of the highly-repetitive central block repeat coding regions and many researchers have addressed this complication by assembling multiple copies of synthetic block repeat domains [9,11]. These block repeat domains, alone and in various combinations with NTD and/or CTD, have been expressed in heterologous prokaryotic and eukaryotic expression systems including, but not limited to, bacteria [12,13], yeast [14], insect cell lines [15,16], and plants [9,17,18,19], albeit with varying levels of success. Most heterologous expression systems, however, have been plagued by low expression levels for a variety of reasons. These include instability of cloned repetitive nucleic acid sequences (rearrangements/deletions, [20]), translational pausing [21,22], depletion of amino acid and/or tRNA pools (due to highly-repetitive protein sequences, [23]), and low solubility [24,25].

Leishmania are single-celled eukaryotic insect vector parasites that naturally secrete various proteins and protein polymers/gels including a high molecular weight phosphoproteoglycan that accumulates in the insect midgut and is the vehicle for parasite transmission to the animal host [26,27]. Another particularly abundant secreted protein is an invertase [28] that has been reported to contribute to the availability of metabolizable sugars from plant-derived polysaccharides present in the sandfly midgut [29]. It has been demonstrated that invertase secretion is directed by an N-terminal signal sequence (SS, [28]), that the signal sequence functions efficiently in secretion of recombinant invertase, and that the SS is absent from the mature protein [28]. Previous work has also shown that the free-living insect vector stage (promastigote) grows rapidly in a simple synthetic medium to high density [30]. Because of the availability of a characterized protein secretion system, relatively simple culture conditions, and the inability of *L*. *tarentola* to infect mammals (the natural host is the gecko), we chose to explore the use of Leishmania as a possible new recombinant expression system for spidroin mimics.

## Materials and methods

### Leishmania strain and vectors

For the purposes of this study, we used *Leishmania tarentolae* which is a species that infects reptiles such as geckos [31]. Recombinant major ampullate spidroins 1 and 2 (rMaSp1 and rMaSp2) coding regions were designed for expression in the Leishmania vector *pKSNEO*. A 6-histidine (His_6_) tag was placed on the C-terminal end of the deduced protein sequence and a secretion signal from *L*. *mexicana* invertase was added to the N-terminal end of the deduced protein sequence. These allowed for secretion and affinity purification of the recombinant protein. Secretion expression of the mini spidroin mimics was chosen for several reasons. First, spidroin proteins are natively secreted into the silk gland. Second, secretion simplifies protein purification. And third, the accumulation of a non-native protein inside the Leishmania cells, that could have serious deleterious consequences, was avoided. The Leishmania used in this expression study were *Leishmania tarentolae J101* (Jena Bioscience). The nomenclature follows Clayton et al. [32]. Plasmid *pKSNEO* was chosen as the expression vector for secretory spidroin mimics based on the previous successful use of *pKSNEO* for recombinant *Leishmania* secretory protein expression [28,33,34]. The shuttle vector *pUC19* (New England Biolabs) was also used in this study to aid in the molecular cloning process.

### Routine cell line maintenance

Leishmania cell lines were routinely passaged in M199 media (Sigma cat #M2520-1L) supplemented with fetal bovine serum (FBS) at a 10% (v/v) final concentration, adenosine, pen/strep, folic acid, hemin, glutamax, and bicarbonate. From a max density culture ~10^8^ cells/mL, 100–200 μL of culture was passaged into 5 mL of media with or without appropriate drug selection.

### Transformation/transfection of bacteria and Leishmania

Electroporation methodologies were used to introduce nucleic acids into both *E*. *coli* [35] for cloning purposes and into Leishmania [28] to generate spidroin mimic cell lines. *E*. *coli* cells were plated on LB+ampicilin plates (100 μg/mL ampicillin) for selection and colonies were screened by mini-plasmid prep isolation [36], restriction enzyme digestion and sequencing. To establish Leishmania cell lines, the drug G418 was added to the M199+10%FBS (fetal bovine serum) growth media at 10 μg/mL initially. Cell lines were passaged routinely and the drug concentration was increased to 50 μg/mL gradually over a one-month period.

#### MaSp1 and MaSp2 mimic constructs

**Fig 1:**
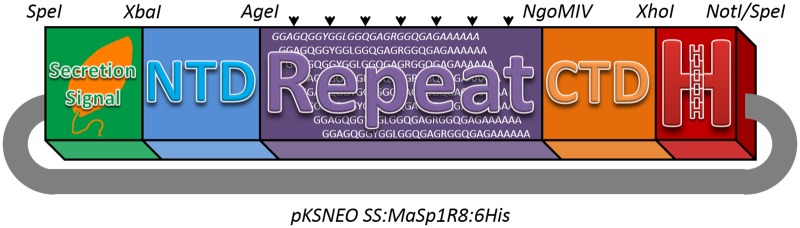
Construct diagram. A diagram highlighting the major landmarks for the construction of *pKSNEO* MaSps. Between the restriction sites SpeI and XbaI a Leishmania secretion signal was devised (Green labeled box). Between the restriction sites XbaI and AgeI, the N-terminal domain (NTD) was inserted (Blue labeled box). Between the restriction sites AgeI and NgoMIV, the repeat domain region was inserted (Purple labeled box). Between the NgoMIV and XhoI restriction sites, the C-terminal domain (CTD) was inserted (Orange labeled box). Lastly, between the restriction sites XhoI and NotI/SpeI a His_6_ tag was inserted (Red labeled box).

The *Nephila clavipes* NTD and CTD sequences used for MaSp1 and MaSp2 expression constructs were previously isolated and described [9,37]. Generation of synthetic MaSp1 and MaSp2 consensus block repeat domains and the assembly of NTD-R#-CTD units (where R# symbolized the number of block repeats) has also been described elsewhere [9,37]. A His_6_ tract followed by an SpeI site was added to the end of the CTD by polymerase chain reaction (PCR). A secretory signal sequence (SS) domain encoded by the *L*. *mexicana* invertase gene (amino acids 1–22,) was amplified by PCR (primers detailed in Table 1) using *pKSNeo*::LmexINV-HA [28] as a template and cloned upstream of the NTD. The entire SS-NTD-R8-CTD-His_6_ units for MaSp1 and MaSp2 were independently cloned into *pKSNEO* as SpeI fragments. This is diagrammatically shown in Fig 1 with the utilized restriction enzymes indicated. Expression and secretion of recombinant protein (including removal of the signal peptide) should result in an N-terminal K-S-R-T-P-G upstream of the native mature MaSp1 or MaSp2 NTD sequence [28].

**Table 1:** Primers used in the construction of plasmids containing *L*. *mexicana* invertase secretion signal. The underlined DNA sequences indicate the addition of restriction sites to the primer for use in the molecular cloning process.

Primer Name	Sequence (5’ to 3’)
F LmexINVSS	GGATCCACTAGTATGCGCCGCGGGGTCATTCTGC
R LmexINVSS	GTCGACACTAGTGCGGCCGCTCTAGACTTTACAAGGGCGCCTGC

### Protein production

To produce protein for fiber production, 500 mL of M199+1% FBS and 50 μg/mL G418 was seeded with 5 mL of culture at max density in a 2 L shake flask. After one week of growth at 26°C and 150 rpm, cell cultures were centrifuged at 3500 rpm (~2000 xg) for 15 minutes to pellet the cells. The liquid supernatant was then run through a column containing 1 mL of Ni beads (Roche Product# 05893682001) by gravity flow, the column was washed and bound protein eluted with elution buffer as directed by Roche protocol. Aliquots of elutions were kept for SDS-PAGE analysis and the remainder of each elution could be dialyzed against 5 mM ammonium bicarbonate. The samples, dialyzed or not, were then frozen at -80°C and subsequently lyophilized to powder (0.08 mBarr, -40°C; Labconco FreeZone 2.5).

### Protein detection using Coomassie and western blot techniques

Protein samples were electrophoresed on 10–12% SDS-polyacrylamide gels and either stained with 0.1% Coomassie Blue R250 or transferred to PVDF membrane.

Spidroin mimic proteins were detected using a 1° rabbit anti-NTD antibody [9], an AP-conjugated 2° goat anti-rabbit/mouse antibody and Lumi-Phos WB alkaline phosphatase solution (Pierce, Cat. No. #34150) for visualization with a Fujifilm LAS-1000plus imager.

### Hand-pulled fiber production

Lyophilized protein powder (~0.25–0.5 g) was then dissolved in 2 mL of a spin solution (80 mM urea, 0.5 mM Tris, 5 mM NaH_2_PO_4_, 10 mM NaCl, pH 5)[38]. Gellan gum solution (0.5% in water) was kept at 55°C to maintain a liquid state. To pull fibers, 10 to 20 μL of protein solution was placed on Parafilm next to 100 to 200 μL of 0.5% gellan gum solution. Then, using forceps, the gum was collided with the protein droplet and fibers were pulled out from the interface. Pulled fibers were allowed to dry at RT on wooden applicator sticks (Fisher Scientific).

### Hand-pulled fiber production using glutaraldehyde as a cross-linking agent

Protein powder (~0.25–0.5 g) was added to a 10% glutaraldehyde solution (100–200 μL) and allowed to react at RT for a few hours to overnight. After the incubation period, 10 μL of the solution was added to 50 μL of spin solution and fibers were pulled as described above using Parafilm.

### Hand-pulled fiber production with ampicillin addition

To add ampicillin to the fiber production methodologies described above, ampicillin was added to the 0.5% gellan gum solution at 10 mg/mL.

### Mechanical property characterization

The mechanical properties of the fibers were determined using tensile tests with a Bruker CETR UMT2 system. Specimens were sectioned from larger fibers using scissors and then mounted onto 9 cm^2^ paper chads with a 1 cm^2^ opening in the center using superglue such that the initial length of the fibers were 1 cm. [39]. Immediately prior to testing, the paper chad was cut to leave the fiber as the sole connection between the top and bottom of the chad. The engineering stress, engineering strain, Young’s modulus and toughness for each fiber were calculated to take into consideration the average specimen diameter.

Before the tensile tests were performed, the general shape, diameter and evenness of the fibers was characterized (Nikon SMZ1500 with a Q Imaging MicroPublisher 3.3 RTV camera, 11.25 X magnification). After tensile testing, the fracture surfaces were imaged using both optical and electron microscopy to inform the analysis of the tensile data with observational break point phenotypes.

### Electron microscopy of pulled fibers

To more accurately assess the fiber surface and breakpoint morphologies, electron microscopy was used. The fibers were attached to a chad at a 45 degree angle such that the break point could be visualized by SEM without a need to tilt. Once attached the samples were coated with platinum by sputter coating for 5 minutes (~5–7 nm thick coat). SEM was performed on an SF4800 High Resolution Scanning Electron Microscope (Hitachi) at the Clemson University Electron Microscopy Facility. Magnification was performed up to 2000X for the images taken.

### Growth inhibition assays

Two assays were used to test the ability of the fibers produced with ampicillin-supplemented gellan gum to inhibit growth of bacteria cells. In one, LB liquid medium was inoculated with a single colony of *E*. *coli* JM109 and was split into two tubes. A fiber produced in the presence of ampicillin was added to one tube and a fiber produced in the absence of ampicillin was added to the other tube (control). After overnight incubation at 37°C (~200 rpm), cultures were observed for bacterial growth. The second inhibition assay used LB solid medium onto which *E*. *coli* JM109 cells were spread. After the plates was spread, fibers produced in the presence or absence of ampicillin were placed on the plates. After overnight incubation at 37°C, the plates were evaluated for growth.

## Results

### Creation of *Leishmania tarentolae pKSNEO*::SS-MaSpR8-His_6_ cell lines

The first goal of this study was to create a cell line of *Leishmania tarentolae* capable of secreting mini-spidroin protein mimics. Two expression cassettes were assembled in *pKSNEO*, one each for MaSp1 and MaSp2, containing eight copies of the respective block repeat domain, as described in the Materials and Methods section (Fig 1). The N-terminus of the protein coding region was modified to include a secretory signal domain from the *Leishmania mexicana* invertase [28] to enable secretion of the spidroin mimics into the culture medium.

### Extraction/purification of recombinant R8 mini-spidroins from culture

**Fig 2:**
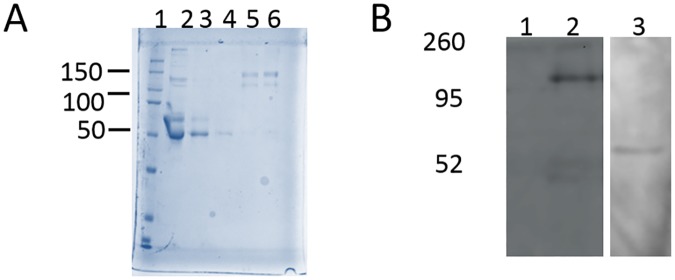
Coomassie Blue and western blot detection of recombinant spidroin mimics. A, Coomassie Blue-stained 10% SDS-PAGE gel analysis of rMaSp2R8 purification steps. Lane 1, molecular weight markers (kDa); Lane 2, culture supernatant; Lane 3, flow through; Lane 4, pooled washes; Lane 5, pooled elutions; Lane 6, column matrix after purification; B, Immuno-detection of rMaSp1R8 and rMaSp2R8 using MaSp NTD-specific antibody. Lane 1, *Leishmania* cells without the addition of any of the *pKSNEO* MaSp vectors; Lane 2, Leishmania cells which express protein from the *pKSNEO* MaSp1R8 vector system; Lane 3, Leishmania cells which express protein from the *pKSNEO* MaSp2R8 vector system. Molecular weight markers are shown to left (kDa).

Successful secretion of mini-spidroin proteins would enable us to put our protein of interest in the culture media liquid. To reduce the amount of unwanted protein within the culture, the media was supplemented with 1% (v/v) FBS instead of the typical 10% added to most routine Leishmania promastigote cultures. To purify the protein, the full 500 mL volume of centrifuged culture supernatant was passed over a single Ni column (1 mL bed volume). After washing, the protein was eluted in a final volume of 5 mLs. Subsequently, the protein eluate was optionally dialyzed and then lyophilized to produce a white powder. Fig 2A shows Coomassie Blue results of a typical purification of recombinant spidroin proteins and indicates the relative amounts of total protein present in collected fractions during the protein purification. As the protein is purified on the column, multimers were detected (lanes 4 and 5). Immuno-detection of protein in the culture supernatant from *L*. *tarentolae* cells transfected with the *pKSNEO* SS:MaSpR8:His_6_ constructs by anti-NTD western blotting after nickel ion: His_6_ tag affinity purification demonstrated proper secretion (Fig 2B).

### Morphology and mechanical properties of the hand-pulled fibers

**Fig 3:**
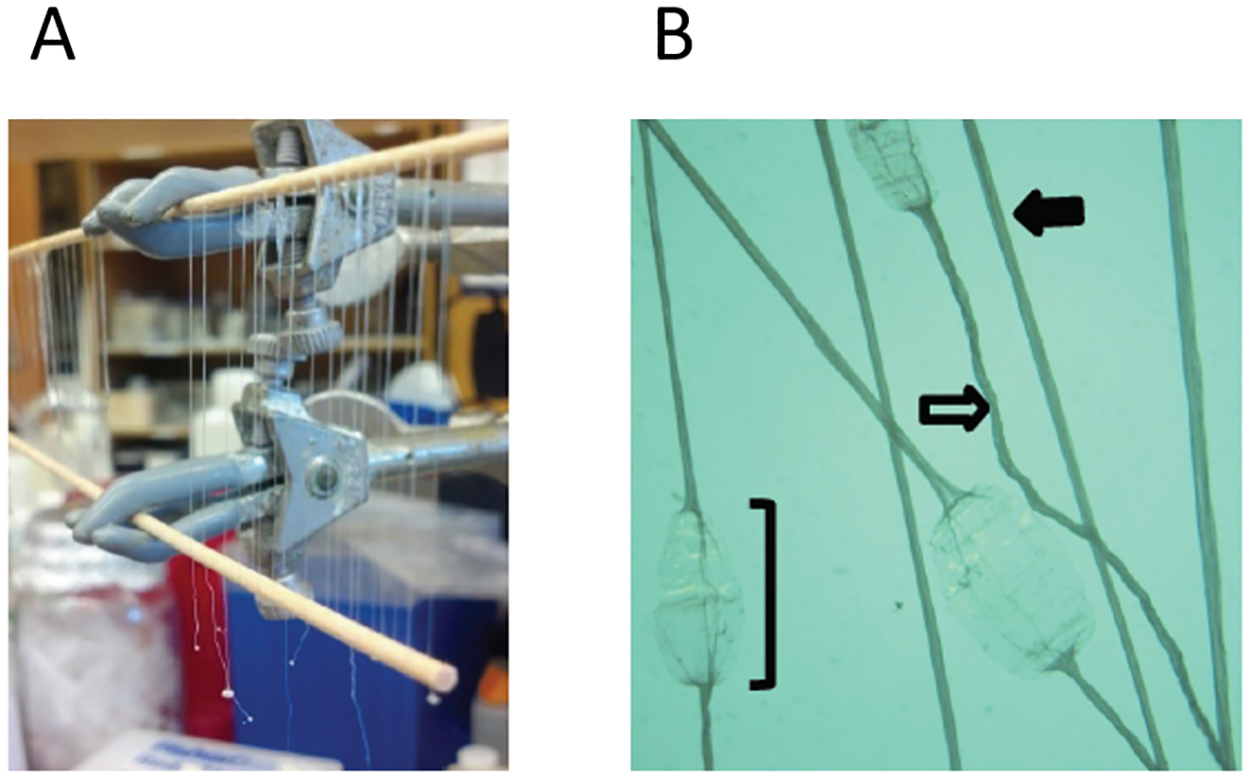
Hand-pulled fiber production. A, a photo of recombinant MaSp fibers drying when attached at both ends; B, a stereoscope image indicating the basic morphology of fibers produced. The solid arrow indicates a fiber that was attached at both ends when dried. The open arrow indicates a fiber that was only attached at one end and allowed to dry while suspended. The bracket indicates an attachment point where a fiber was dried and flattened due to surface adherence.

Fibers (Fig 3) were successfully produced using interfacial polyion complexation based on Meier and Welland (2007)[40]. The different treatments used for fiber pulling are summarized in Table 2. It was determined that straight fibers could be produced if the fibers were dried with attachment points at the top and bottom. If allowed to dry while hanging with only one attachment point, the fiber would twist and curl (Fig 3B). Optical analysis of the individual hand-pulled fibers showed that the average diameters ranged from 20–150 μm among the fibers. The variation along a single fiber was much smaller, suggesting that the variance in fiber diameter could be strongly influenced by variability in manual pulling style and speed for each fiber.

**Table 2:** Summary of spidroin-like fibers produced. This table shows the naming scheme used within this manuscript. Check marks indicate the presence of gellan layering and additives in the fiber pulling process.

Abbreviated Fiber Name	Gellan layering	Glutaraldehyde pretreated	Ampicillin addition to gellan
MaSp1R8 Only	✓		
MaSp2R8 Only	✓		
MaSp1R8 & Glut	✓	✓	
MaSp2R8 & Glut	✓	✓	
MaSp1R8 & Amp	✓		✓
MaSp2R8 & Amp	✓		✓

**Fig 4:**
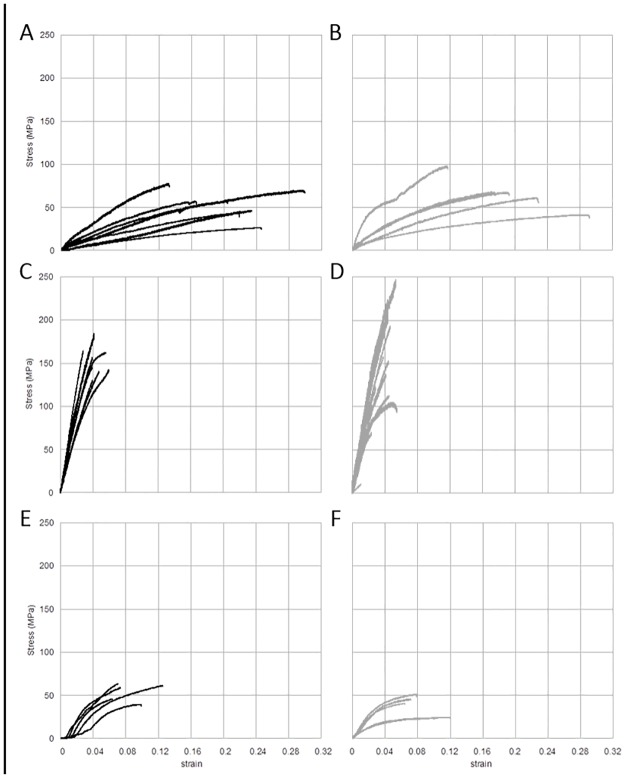
Stress/Strain curves for MaSp1R8 and MaSp2R8 hand-pulled fibers. Stress/Strain curves produced from tensile measurement of hand-pulled fibers. A, Sp1R8 only; B, Sp2R8 only; C, Sp1R8 plus glutaraldehyde; D, Sp2R8 plus glutaraldehyde; E, Sp1R8 plus ampicillin; F, Sp2R8 plus ampicillin. Individual lines in each panel represent replicates.

The tensile results for each sample type can be used to compare the influence of each processing variation on fiber mechanical properties (Fig 4). The number of specimens varied between each fiber type since specimens showing evidence of ‘slip’ from the superglue during testing were excluded. In general, fibers made from the MaSp1 and MaSp2 proteins (Fig 4A and 4B), displayed much greater elongation prior to failure than equivalent fibers made with the crosslinking agent glutaraldehyde or with ampicillin (Fig 4C–4F). Table 3 quantifies this difference and the other tensile parameters (Young’s modulus, breaking stress, breaking strain and toughness).

**Table 3:** Tensile testing of spidroin-like fibers. Mean values for tensile properties and the corresponding standard deviations (STDEV) were recorded. Units for each parameter in the table are described in the column headers. Data for *N*. *clavipes* major ampullate (MA) silk comes from Gosline et al. [5].

Sample	Sample Number	Young's Modulus (MPa)		Breaking Stress (MPa)		Breaking Strain (unitless)		Toughness (J/cm^3)	
		mean	STDEV	mean	STDEV	mean	STDEV	mean	STDEV
Sp1R8 Only	7	572	339	53	17	0.21	0.05	6.63	2.89
Sp1R8 & Amp	5	1465	671	53	11	0.08	0.02	2.56	1.15
Sp1R8 & Glut	9	4587	854	148	21	0.04	0.01	3.45	1.35
Sp2R8 Only	5	1220	853	67	21	0.20	0.07	8.07	0.55
Sp2R8 & Amp	5	1067	342	37	13	0.09	0.02	2.15	0.40
Sp2R8 & Glut	14	4500	1347	142	59	0.04	0.01	3.35	1.79
*N*. *clavipes* MA Silk		22000		1300		0.12		80	

### Fracture surface characterization

**Fig 5:**
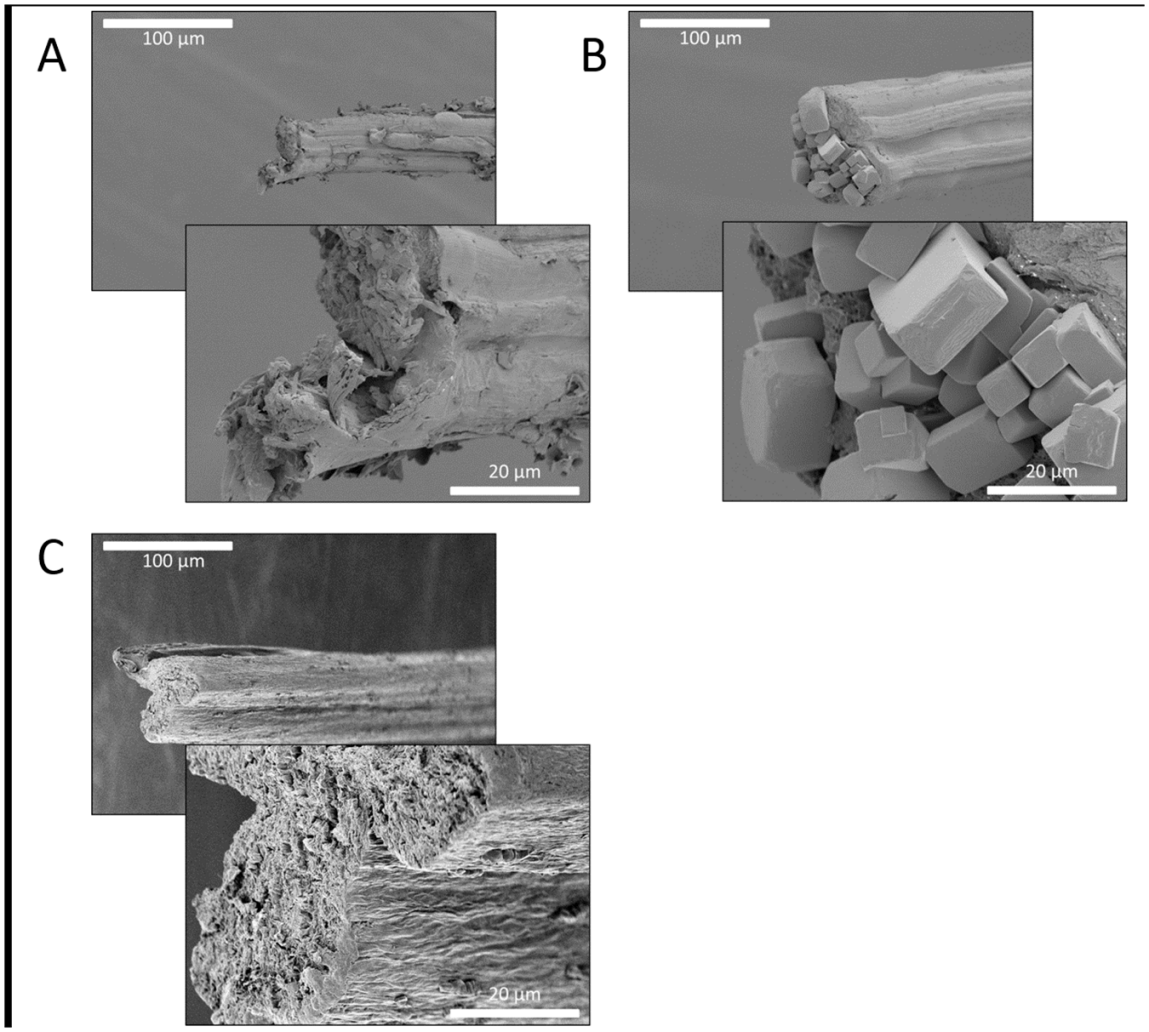
SEM break point observations of rMaSp1R8 fibers. Panels A, rMaSp1R8 fiber; Panels B, rMaSp1R8 fiber pretreated with ampicillin before fiber pulling; Panels C, rMaSp1R8 fiber pretreated with glutaraldehyde before fiber pulling. Scale bars show 100 μm or 20 μm lengths.

**Fig 6:**
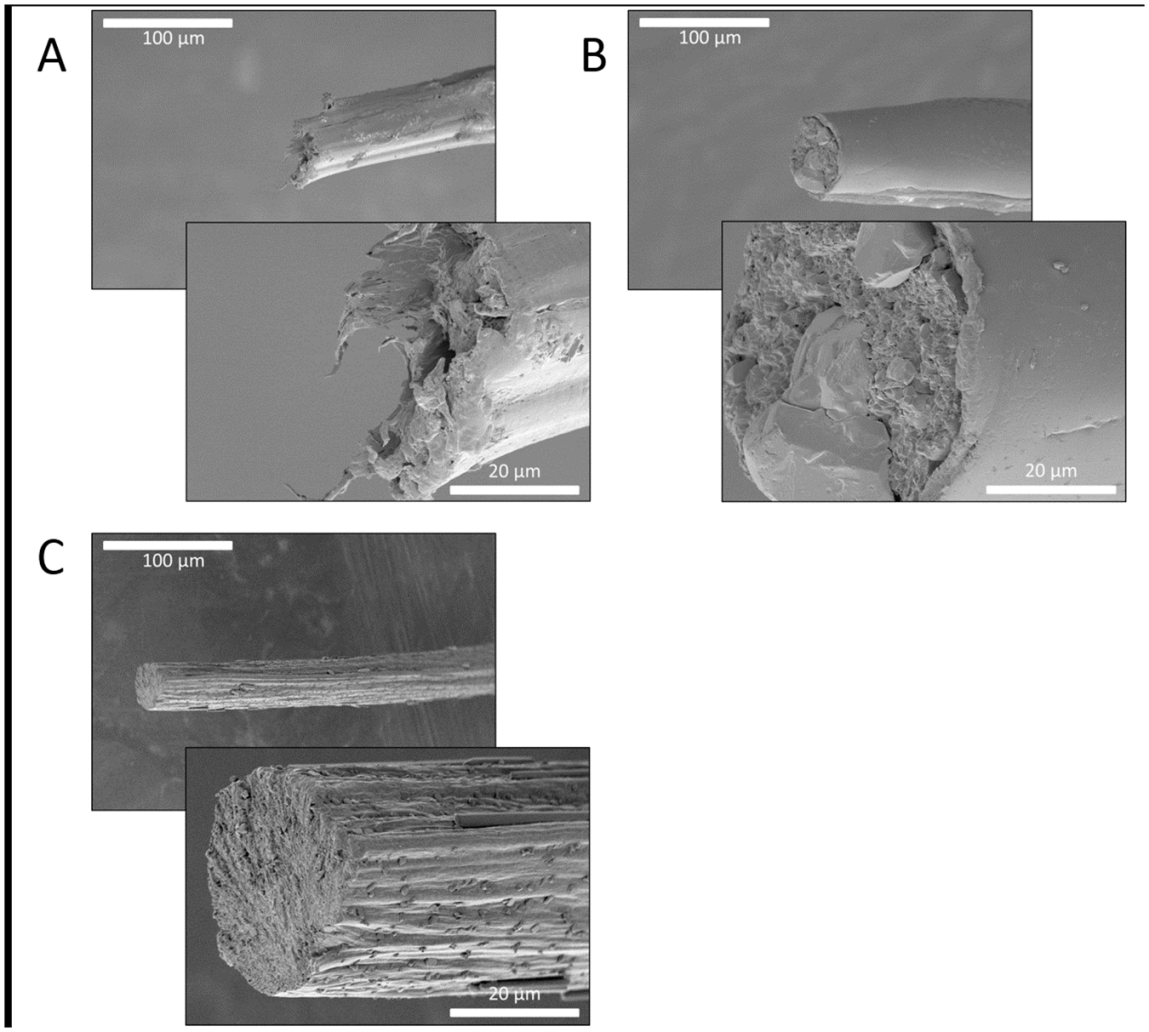
SEM break point observations of rMaSp2R8 fibers. Panels A, rMaSp2R8 fiber; Panels B, rMaSp2R8 fiber pretreated with ampicillin before fiber pulling; Panels C, rMaSp2R8 fiber pretreated with glutaraldehyde before fiber pulling. Scale bars show 100 μm or 20 μm lengths.

The break points from the tensile tests were observed under a microscope. The breakpoint morphology varied in appearance under stereoscope microscopy indicating that fibers had different breaking mechanisms. Electron microscopy revealed a clean/smooth break for the glutaraldehyde-treated samples and a torn break for the untreated samples (Figs 5 and 6). Fig 5 contains representative SEM images of break points for the rMaSp1R8 fibers produced while Fig 6 contains representative SEM images of break points for the rMaSp2R8 fibers. The smooth breaks are consistent with the proposed hierarchical structure of spidroin proteins in fibers [41]. The clean break morphology of the glutaraldehyde samples indicates that these fibers were much more brittle than the untreated fibers. Additionally, it has been observed that the rMaSp1R8 fibers tend to form a fiber shaped like a celery stalk (C-shaped) while the rMaSp2R8 fibers tend to be round in shape. Salt crystals could be observed on the outside and inside of many of the samples in which dialysis was not performed and in the ampicillin treated fibers (Figs 5 and 6).

### Inhibition of bacterial growth with ampicillin-enhanced fibers

**Fig 7:**
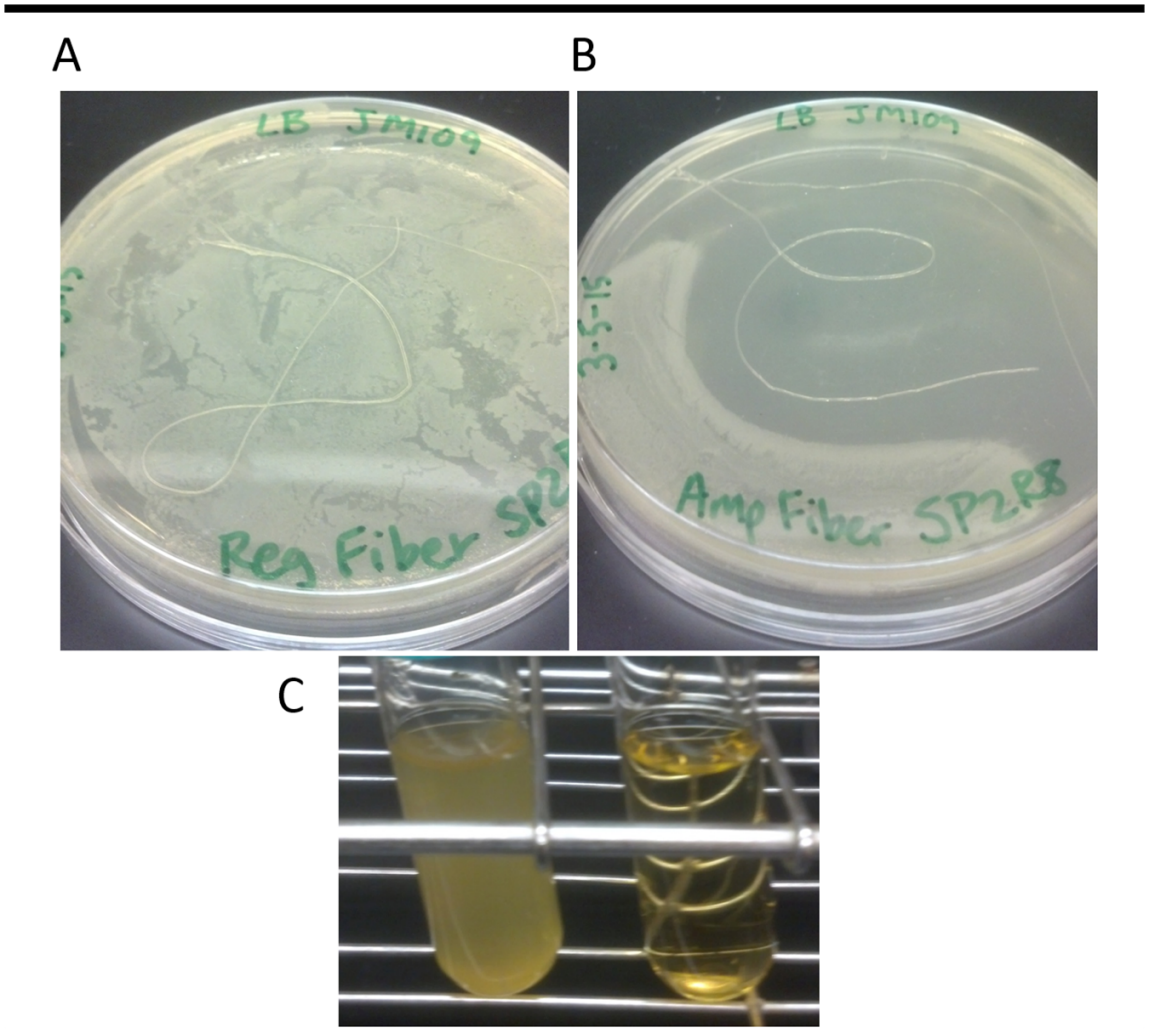
Bacterial growth inhibition assays. Inhibition zone assay with Leishmania derived fibers treated with or without ampicillin. Ampicillin was added to gellan gum solution at 10 mg/mL prior to pulling fibers. Fibers were allowed to air dry for several hours and then placed on LB-agar plates spread with *E*. *coli* bacteria. A, an inhibition plate assay using a recombinant MaSp2R8 fiber; B, an inhibition plate assay using a recombinant MaSp2R8 fiber which was treated with ampicillin during the fiber production stage; C, liquid culture showing inhibition of bacterial growth when grown in the presence of a recombinant MaSp2R8 fiber treated with ampicillin during the production process.

Two *in vitro* approaches, one using solid medium, one using liquid, were devised to assess whether the ampicillin-enhanced recombinant silk fiber mimics could inhibit growth of bacteria. Both approaches would determine whether the antibiotic remained active as a component of the fiber and if the antibiotic would be able to disperse from the fiber. In the first approach, spidroin fiber containing ampicillin was placed on an LB agar plate spread with *E*. *coli* bacteria. The LB agar plate approach showed a definitive zone of inhibition around the ampicillin-enhanced fibers when compared to control fibers (Fig 7A and 7B). A similar result was found using liquid culture inoculated with *E*. *coli*. A single fiber proved very effective in inhibiting bacterial growth (Fig 7C).

## Discussion

It is extremely difficult to get large quantities of spider silks for any commercial application. Spiders do not adapt well to captivity. In taking a molecular approach, the problems of spider feeding and cannibalism are eliminated yet, new challenges arise. Spider silk genetic sequences are difficult to clone using general molecular biology techniques due to their size and repeat units. As a result, we and other groups have designed mini spidroin mimics of varying composition and size. We incorporate all three of the dragline silk domains in our design: NTD, Block Repeat Region, and CTD.

In nature, species of Leishmania cause Leishmaniasis, a global disease manifested as cutaneous, mucocutaneous and visceral diseases in humans and other animals [42]. It is spread by the bite of parasite-infected sand flies. During the life cycle of the parasite, it is alternately present in two hostile micorenvironments, the sand fly midgut (insect host compartment, nutrient-poor, pH ~9.0,[43]) and macrophage phagolysosomes (animal host compartment, nutrient-rich, pH ~5.0–6.0, [44]). Promastigotes, the insect vector stage, reside and divide within the midgut of the sand fly and so are dependent on the feeding practices of the sand fly for nutrients. Nutrients are abundant immediately after a blood meal (about every seven days) between which the fly relies on plant-derived sugar meals. These complex carbohydrates (e.g. sucrose) must be broken down into simpler sugars for energy utilization and the promastigotes secrete large quantities of an invertase that facilitates this process [28]. Promastigotes also secrete abundant amounts of phosphoproteoglycans that eventually accumulate to form a gelatinous plug that closes off the midgut. In order to properly feed, the sand fly must expel the infectious plug, resulting in the introduction of parasites into the bite wound [45,46]. The ability of Leishmania parasites to secrete large quantities of various proteins, the availability of a characterized secretion system [28], and the wide range of acceptable growth conditions, make Leishmania an ideal candidate for large-scale spider silk protein production.

*Leishmania tarentolae* was chosen as the expression system due to its fast growth, high cell density and simple media formulation. In this way, the rMaSp1 and rMaSp2 can be grown at RT to 26°C and secreted into the medium similar to natural conditions of silk production by spiders. Secretion also simplified protein preparation/purification. After a simple centrifugation step, the culture media is allowed to pass through a nickel ion affinity column to purify the recombinant protein. The protein was prepared for fiber production with dialysis and lyophilization clean up steps to yield powdered recombinant protein.

Our hand-pulling technique proved effective at producing fibers. However, the recombinant fibers were significantly thicker than native fibers. Tensile measurements of each of the produced fibers confirmed our initial hypothesis that addition of glutaraldehyde or ampicillin would alter the fiber properties.

Glutaraldehyde has been shown to act as a non-zero-length cross-linking agent that can alter the mechanical properties of a variety of protein-based biomaterials [47,48] and remains one of the most widely used cross-linking agents due to availability and practicality [49]. In studies with gelatin membranes, glutaraldehyde crosslinking enhanced mechanical stability and decreased sensitivity to *in* enzymatic degradation [48]. Similar enhancement of mechanical stability and resistance to enzymatic degradation by glutaraldehyde treatment have been found with amniotic membranes [47]. However, glutaraldehyde treatment of biopolymers typically compromises their biocompatibility as evidenced by decreases in cell proliferation and elevated expression of IL-6 in cytokine bioassays [47,48].

For this study, glutaraldehyde addition increased both initial stiffness (Young’s modulus) and strength (breaking stress). The toughness of the fiber, however, decreased by at least 50% due to a reduction in extensibility (breaking strain). Reduced breaking strain is also consistent with the increase observed in initial stiffness. SEM revealed that the break points of the fibers containing glutaraldehyde were extremely smooth compared to the protein alone fibers (compare Figs 5A with 5C and 6A with 6C), indicative of a strong yet brittle fiber.

Ampicillin addition gave the recombinant spidroin fibers the ability to inhibit bacterial growth (Fig 7) but decreased the overall toughness due to a reduction in strength and/or extensibility. This reduction in toughness may be explained by a build-up of crystalline structures, presumably ampicillin and other salts, leading to the creation of weak points in the fiber (Figs 5B and 6B).

So far, the results have been promising. One major concern with this system is protein yield for commercial scale up applications. Currently, we are investigating the use of bioreactors as a way to increase yield through controlled cellular proliferation. Additionally, more controlled methods for fiber production may lead to more controlled widths and lengths. Such methods of future investigation will include electrospinning and alcohol coagulation baths [50,51]. It is our intent to achieve fiber thickness closer to native spider silk (2–4 μm); however, larger and smaller widths could still yield fibers suitable for certain applications. We are also developing expression constructs containing increased numbers of the block repeat. These extended fiber mimics will be interesting to test and a set of extended repeat unit lengths should give rise to a range of tensile properties to choose from for biomedical, textile, and novel uses.

This study not only demonstrates that *Leishmania tarentolae* is a viable option for the production of recombinant spidroin protein mimics for fiber assembly but once purified, the addition of small molecules to the fiber pulling process can yield an array of fibers with varying properties for future beneficial products.

## References

[1] Lucas F, Rudall KM. Extracellular fibrous proteins: the silks. Comprehensive Biochemistry 1968;26:475–558.

[2] Lucas F. Spiders and their silks. Discovery 1964;25:20–26.

[3] Hinman MB, Jones JA, Lewis RV. Synthetic spider silk: a modular fiber. Trends Biotechnol 2000 9;18(9):374–379. 10942961 10.1016/s0167-7799(00)01481-5

[4] Gosline JM, DeMont ME, Denny MW. The structure and properties of spider silk. Endeavor 1986;10(1):37–43.

[5] Gosline JM, Guerette PA, Ortlepp CS, Savage KN. The mechanical design of spider silks: from fibroin sequence to mechanical function. J Exp Biol 1999 12;202(Pt 23):3295–3303. 10562512 10.1242/jeb.202.23.3295

[6] Hinman MB, Lewis RV. Isolation of a clone encoding a second dragline silk fibroin. Nephila clavipes dragline silk is a two-protein fiber. J Biol Chem 1992 9 25;267(27):19320–19324. 1527052

[7] Xu M, Lewis RV. Structure of a protein superfiber: spider dragline silk. Proc Natl Acad Sci U S A 1990 9;87(18):7120–7124. 2402494 10.1073/pnas.87.18.7120 PMC54695

[8] Gaines WA, Sehorn MG, Marcotte WR Jr. Spidroin N-terminal domain promotes a pH-dependent association of silk proteins during self-assembly. J Biol Chem 2010 12 24;285(52):40745–40753. 10.1074/jbc.M110.163121 20959449 PMC3003374

[9] Peng CA, Russo J, Gravgaard C, McCartney H, Gaines W, Marcotte WR Jr. Spider silk-like proteins derived from transgenic Nicotiana tabacum. Transgenic Res 2016 3 30. 10.1007/s11248-016-9949-1 27026165

[10] Vollrath F. Biology of spider silk. Int J Biol Macromol 1999 Mar-Apr;24(2–3):81–88. 10342751 10.1016/s0141-8130(98)00076-2

[11] Teule F, Cooper AR, Furin WA, Bittencourt D, Rech EL, Brooks A, et al A protocol for the production of recombinant spider silk-like proteins for artificial fiber spinning. Nat Protoc 2009;4(3):341–355. 10.1038/nprot.2008.250 19229199 PMC2720753

[12] Arcidiacono S, Mello C, Kaplan D, Cheley S, Bayley H. Purification and characterization of recombinant spider silk expressed in Escherichia coli. Appl Microbiol Biotechnol 1998 1;49(1):31–38. 9487707 10.1007/s002530051133

[13] Heidebrecht A, Eisoldt L, Diehl J, Schmidt A, Geffers M, Lang G, et al Biomimetic fibers made of recombinant spidroins with the same toughness as natural spider silk. Adv Mater 2015 4 1;27(13):2189–2194. 10.1002/adma.201404234 25689835

[14] Fahnestock SR, Bedzyk LA. Production of synthetic spider dragline silk protein in Pichia pastoris. Appl Microbiol Biotechnol 1997;47:33–39. 9035408 10.1007/s002530050884

[15] Wen H, Lan X, Zhang Y, Zhao T, Wang Y, Kajiura Z, et al Transgenic silkworms (Bombyx mori) produce recombinant spider dragline silk in cocoons. Mol Biol Rep 2010 4;37(4):1815–1821. 10.1007/s11033-009-9615-2 19633923

[16] Zhang Y, Hu J, Miao Y, Zhao A, Zhao T, Wu D, et al Expression of EGFP-spider dragline silk fusion protein in BmN cells and larvae of silkworm showed the solubility is primary limit for dragline proteins yield. Mol Biol Rep 2008 9;35(3):329–335. 10.1007/s11033-007-9090-6 17525867

[17] Barr LA, Fahnestock SR, and Yang J. Production and purification of recombinant DP1B silk -like protein in plants. Mol Breed 2004;13:345–356.

[18] Hauptmann V, Weichert N, Rakhimova M, Conrad U. Spider silks from plants—a challenge to create native-sized spidroins. Biotechnol J 2013 10;8(10):1183–1192. 10.1002/biot.201300204 24092675

[19] Scheller J, Guhrs KH, Grosse F, Conrad U. Production of spider silk proteins in tobacco and potato. Nat Biotechnol 2001 6;19(6):573–577. 10.1038/89335 11385464

[20] Sallach RE, Conticello VP, Chaikof EL. Expression of a recombinant elastin-like protein in pichia pastoris. Biotechnol Prog 2009 Nov-Dec;25(6):1810–1818. 10.1002/btpr.208 19827084 PMC2796711

[21] Zama M. Correlation between mRNA structure of the coding region and translational pauses. Nucleic Acids Symp Ser 1999;(42)(42):81–82. 10780389 10.1093/nass/42.1.81

[22] Zama M. Translational pauses during the synthesis of proteins and mRNA structure. Nucleic Acids Symp Ser 1997;(37)(37):179–180. 9586058

[23] Xia XX, Qian ZG, Ki CS, Park YH, Kaplan DL, Lee SY. Native-sized recombinant spider silk protein produced in metabolically engineered Escherichia coli results in a strong fiber. Proc Natl Acad Sci U S A 2010 8 10;107(32):14059–14063. 10.1073/pnas.1003366107 20660779 PMC2922564

[24] Huemmerich D, Helsen CW, Quedzuweit S, Oschmann J, Rudolph R, Scheibel T. Primary structure elements of spider dragline silks and their contribution to protein solubility. Biochemistry 2004 10 26;43(42):13604–13612. 10.1021/bi048983q 15491167

[25] Ittah S, Barak N, Gat U. A proposed model for dragline spider silk self-assembly: insights from the effect of the repetitive domain size on fiber properties. Biopolymers 2010;93(5):458–468. 10.1002/bip.21362 20014164

[26] Rogers ME. The role of Leishmania proteophosphoglycans in sand fly transmission and infection of the mammalian host. Front Microbiol 2012 6 28;3:223 10.3389/fmicb.2012.00223 22754550 PMC3384971

[27] Rogers ME, Corware K, Muller I, Bates PA. *Leishmania infantum* proteophosphoglycans regurgitated by the bite of its natural sand fly vector, Lutzomyia longipalpis, promote parasite establishment in mouse skin and skin-distant tissues. Microbes Infect 2010 10;12(11):875–879. 10.1016/j.micinf.2010.05.014 20561596

[28] Lyda TA, Joshi MB, Andersen JF, Kelada AY, Owings JP, Bates PA, et al A unique, highly conserved secretory invertase is differentially expressed by promastigote developmental forms of all species of the human pathogen, Leishmania. Mol Cell Biochem 2015 6;404(1–2):53–77. 10.1007/s11010-015-2366-6 25763714 PMC4417071

[29] Jacobson RL, Studentsky L, Schlein Y. Glycolytic and chitinolytic activities of Phlebotomus papatasi (Diptera: Psychodidae) from diverse ecological habitats. Folia Parasitol (Praha) 2007 11;54(4):301–309. 18303772 10.14411/fp.2007.039

[30] Fritsche C, Sitz M, Weiland N, Breitling R, Pohl HD. Characterization of the growth behavior of *Leishmania tarentolae*: a new expression system for recombinant proteins. J Basic Microbiol 2007 10;47(5):384–393. 10.1002/jobm.200710111 17910102

[31] Elwasila M. *Leishmania tarentolae* Wenyon, 1921 from the gecko Tarentola annularis in the Sudan. Parasitol Res 1988;74(6):591–592. 3194372 10.1007/BF00531640

[32] Clayton C, Adams M, Almeida R, Baltz T, Barrett M, Bastien P, et al Genetic nomenclature for Trypanosoma and Leishmania. Mol Biochem Parasitol 1998 11 30;97(1–2):221–224. 9879900 10.1016/s0166-6851(98)00115-7

[33] Charest H, Zhang WW, Matlashewski G. The developmental expression of *Leishmania donovani* A2 amastigote-specific genes is post-transcriptionally mediated and involves elements located in the 3'-untranslated region. J Biol Chem 1996 7 19;271(29):17081–17090. 8663340 10.1074/jbc.271.29.17081

[34] Ghedin E, Charest H, Zhang WW, Debrabant A, Dwyer D, Matlashewski G. Inducible expression of suicide genes in *Leishmania donovani* amastigotes. J Biol Chem 1998 9 4;273(36):22997–23003. 9722523 10.1074/jbc.273.36.22997

[35] BTX. Protocol 0013 Electro Cell Manipulator™ ECM^®^600/630 ELECTROPORATION PROTOCOL E. coli DH5α, DH1. BTX Division of Genetronics 2001:1–2.

[36] Sambrook J, Fritsch EF, Maniatis T. Molecular Cloning: A Laboratory Manual, 2nd Ed Cold Spring Harbor Laboratory Press, Cold Spring Harbor, NY 1989:7.43–7.45.

[37] Gaines WA 4th, Marcotte WR Jr. Identification and characterization of multiple Spidroin 1 genes encoding major ampullate silk proteins in Nephila clavipes. Insect Mol Biol 2008 9;17(5):465–474. 10.1111/j.1365-2583.2008.00828.x 18828837 PMC2831225

[38] Arcidiacono S, Welsh EA, Soares JW. Aqueous-based spinning of fibers from self-assembling structural proteins. Methods Mol Biol 2013;996:43–59. 10.1007/978-1-62703-354-1_3 23504417

[39] Żurek W, Kocik M, Całka W, Jakubczyk J. Tensile properties of carbon fibres. Fibre Science and Technology 1981;15(3):223–234.

[40] Meier C, Welland ME. Wet-spinning of amyloid protein nanofibers into multifunctional high-performance biofibers. Biomacromolecules 2011 10 10;12(10):3453–3459. 10.1021/bm2005752 21859156

[41] Xu G, Gong L, Yang Z, Liu XY. What makes spider silk fibers so strong? From molecular-crystallite network to hierarchical network structures. Soft Matter 2014;10:2116–2123. 10.1039/c3sm52845f 24652059

[42] UNICEF/UNDP/World Bank/World Health Organization. Control of the leishmaniases: report of a meeting of the WHO Expert Committee on the Control of Leishmaniases. UNICEF/ UNDP/World Bank/World Health Organization, Geneva 2010.

[43] Fazito do Vale V, Pereira MH, Gontijo NF. Midgut pH profile and protein digestion in the larvae of Lutzomyia longipalpis (Diptera: Psychodidae). J Insect Physiol 2007 11;53(11):1151–1159. 10.1016/j.jinsphys.2007.06.005 17659300

[44] McConville MJ, de Souza D, Saunders E, Likic VA, Naderer T. Living in a phagolysosome; metabolism of Leishmania amastigotes. Trends Parasitol 2007 8;23(8):368–375. 10.1016/j.pt.2007.06.009 17606406

[45] Stierhof YD, Bates PA, Jacobson RL, Rogers ME, Schlein Y, Handman E, et al Filamentous proteophosphoglycan secreted by Leishmania promastigotes forms gel-like three-dimensional networks that obstruct the digestive tract of infected sandfly vectors. Eur J Cell Biol 1999 10;78(10):675–689. 10.1016/S0171-9335(99)80036-3 10569240

[46] Stierhof YD, Ilg T, Russell DG, Hohenberg H, Overath P. Characterization of polymer release from the flagellar pocket of *Leishmania mexicana* promastigotes. J Cell Biol 1994 4;125(2):321–331. 8163549 10.1083/jcb.125.2.321 PMC2120037

[47] Lai JY. Interrelationship between cross-linking structure, molecular stability, and cytocompatibility of amniotic membranes cross-linked with glutaraldehyde of varying concentrations. RSC Adv 2014;4:18871–18880.

[48] Lai JY, Li YT. Evaluation of cross-linked gelatin membranes as delivery carriers for retinal sheets. Mater Sci Eng C 2010;30:677–685.

[49] Reddy N, Reddy R, Jiang Q. Crosslinking biopolymers for biomedical applications. Trends Biotechnol 2015 6;33(6):362–369. 10.1016/j.tibtech.2015.03.008 25887334

[50] Teule F, Furin W, Cooper A, Duncan J, Lewis R. Modifications of spider silk sequences in an attempt to control the mechanical properties of the synthetic fibers. Journal of Material Science 2007;42(21):8974–8985.

[51] Zhou S, Peng H, Yu X, Zheng X, Cui W, Zhang Z, et al Preparation and characterization of a novel electrospun spider silk fibroin/poly(D,L-lactide) composite fiber. J Phys Chem B 2008 9 11;112(36):11209–11216. 10.1021/jp800913k 18710278

